# Impact of malnutrition evaluated by the mini nutritional assessment on the prognosis of acute hospitalized older adults

**DOI:** 10.3389/fnut.2022.1046985

**Published:** 2023-01-05

**Authors:** Min-gu Kang, Jung-Yeon Choi, Hyun-Jung Yoo, Si-Young Park, Yoonhee Kim, Ji Yoon Kim, Sun-wook Kim, Cheol-Ho Kim, Kwang-il Kim

**Affiliations:** ^1^Department of Internal Medicine, Chonnam National University Bitgoeul Hospital, Gwangju, Republic of Korea; ^2^Department of Internal Medicine, Seoul National University Bundang Hospital, Seongnam-si, Republic of Korea; ^3^Department of Nursing, Seoul National University Bundang Hospital, Seongnam-si, Republic of Korea; ^4^Department of Pharmacy, Seoul National University Bundang Hospital, Seongnam-si, Republic of Korea; ^5^Department of Nutrition Care Service, Seoul National University Bundang Hospital, Seongnam-si, Republic of Korea; ^6^Department of Internal Medicine, Seoul National University College of Medicine, Seoul, Republic of Korea

**Keywords:** nutrition, discharge location, geriatric syndrome, mini nutritional assessment, serum albumin

## Abstract

**Background:**

Malnutrition is prevalent among hospitalized older patients. Therefore, this study aimed to investigate the association between nutritional status [assessed using the Mini Nutritional Assessment (MNA) and serum albumin levels] and adverse outcomes in hospitalized older patients. We also aimed to compare the predictive utility of our findings.

**Methods:**

This retrospective cohort study was conducted between January 2016 and June 2020. In total, 808 older patients (aged ≥ 65 years, mean age 82.8 ± 6.70 years, 45.9% male) admitted to the acute geriatric unit were included in our sample. Comprehensive geriatric assessments, including the MNA, were performed. Malnutrition and risk of malnutrition were defined as MNA < 17, albumin < 3.5 g/dL and 17 ≤ MNA ≤ 24, 3.5 g/dL ≤ albumin < 3.9 g/dL, respectively. The primary outcome was that patients could not be discharged to their own homes. The secondary outcomes were overall all-cause mortality, 3-month all-cause mortality, and incidence of geriatric syndrome, including delirium, falls, and newly developed or worsening pressure sores during hospitalization.

**Results:**

Poor nutritional status was associated with older age; female sex; admission from the emergency room; high risk of pressure sores and falls; lower physical and cognitive function; higher depressive score; and lower serum albumin, protein, cholesterol, and hemoglobin levels. In the fully adjusted model, malnutrition assessed using the MNA predicted discharge to nursing homes or long-term care hospitals [odds ratio (OR) 5.822, 95% confidence interval (CI): 2.092–16.199, *P* = 0.001], geriatric syndrome (OR 2.069, 95% CI: 1.007–4.249, *P* = 0.048), and 3-month mortality (OR 3.519, 95% CI: 1.254–9.872, *P* = 0.017). However, malnutrition assessed using albumin levels could only predict 3-month mortality (OR 3.848, 95% CI: 1.465–10.105, *P* = 0.006). The MNA predicted 3-month mortality with higher precision than serum albumin levels (*P* = 0.034) when comparing the areas under the receiver operating characteristic curve.

**Conclusion:**

Nutritional risk measured by the MNA was an independent predictor of various negative outcomes in hospitalized older patients. Poor nutritional status assessed by serum albumin levels, the most widely used biochemical marker, could predict mortality, but not the development of geriatric syndrome or discharge location reflecting functional status.

## 1. Introduction

Malnutrition is an imbalance between food intake and body requirements, which results in altered metabolism, impaired function, and loss of body mass (1, 2). Malnutrition is common in older adults (3). People eat less and make different food choices as they age (4). Age-related physiological changes, including slower gastric emptying, changes in hormonal responses, and altered taste and smell, can contribute to decreased food intake (5). Other factors such as marital status, income, education, and socioeconomic status may also influence eating habits among older adults (6).

Studies have shown that malnutrition has serious implications for recovery from disease and is associated with increased morbidity and mortality (2, 7). Malnourished older adults tend to have higher rates of complications and infections, as well as longer hospital stays (8, 9).

Poor nutritional status is also associated with geriatric syndrome (10). Undernutrition is a cornerstone in the concept of the cycle of frailty, a self-aggravating cycle of negative energy balance, and the cause of decreased physical activity and a further decline in physical performance (11, 12). Previous studies have confirmed that malnutrition contributes to the development of delirium and pressure sores in hospitalized older patients (13, 14). Additionally, malnutrition at the time of hospital admission is a major risk factor for in-hospital falls (15).

The likelihood of patients being alive and in their own homes after hospital discharge is an important goal in the care of hospitalized older patients (16). After acute hospitalization, frail older adults are more likely to be admitted to nursing facilities due to their dependency on assistance with activities of daily living (ADL) (17, 18). However, institutionalization often leads to a more rapid deterioration of function due to the perpetual bedridden state (19, 20). While the location of discharge after acute geriatric hospitalization is an important issue in older patients, studies on the association between nutritional status and discharge location are limited.

As comprehensive nutritional assessment is complex and time consuming, several screening tools are used to assess nutritional status. For instance, the Mini Nutritional Assessment (MNA) is a validated test recommended for nutritional screening in older populations and has been widely used in different clinical settings (21, 22). The MNA is a practical, non-invasive tool that allows rapid evaluation of the nutritional status of older adults (23, 24). Various studies on the association between malnutrition and clinical outcomes in hospitalized older adults using the MNA have been conducted (25, 26). The MNA was useful for predicting frailty in hospitalized older patients (27), and lower MNA scores were significant predictors of post-discharge emergency department visits (28) and mortality outcomes (29–31). However, in another study, malnutrition as diagnosed with the MNA at admission failed to predict long-term mortality in older inpatients (26).

Despite its clinical significance, studies on the predictive role of the MNA in the hospital course and outcomes of acutely hospitalized older patients are scarce. In particular, there is a paucity of studies evaluating the prognostic prediction of the MNA by comparison with widely used biochemical markers. Therefore, we aimed to analyze the efficacy of the MNA in predicting geriatric syndrome, discharge location, and mortality in patients admitted to the Geriatric Center of a university hospital and compared the prognostic utility of the MNA and serum albumin levels.

## 2. Materials and methods

### 2.1. Study design and participants

This retrospective cohort study was conducted in Seoul National University Bundang Hospital between January 1, 2016 and June 30, 2020. The study protocol was reviewed and approved by the Institutional Review Board of Seoul National University Bundang Hospital. The requirement for informed consent was waived because of the retrospective nature of the study, and it was impossible to obtain consent from each participant who had already been discharged (B-2206-760-106).

Older patients (aged ≥ 65 years) who were admitted to the Geriatric Center from their own home and underwent comprehensive geriatric assessment (CGA) were included. If patients were admitted more than once, only the data corresponding to the last admission were analyzed. Patients with incomplete nutritional assessments were excluded.

### 2.2. Patient assessment

Baseline patient characteristics, including demographic, anthropometric, laboratory data, and admission site (emergency room or outpatient clinic) were retrieved from the electronic medical record systems. The risk of fall and pressure score were evaluated using the Hendrich II Fall Risk Model and Braden Scale (for predicting pressure ulcer risk in routine nursing practice) (32, 33). The CGA, a multidisciplinary and interdisciplinary process, is the accepted gold standard for the care of older, frail, hospitalized patients. The CGA consists of medication assessment, comorbidity, muscle strength, cognitive function, depression, and nutrition. Medication lists were reviewed by a pharmacist, and a potentially inappropriate medication (PIM) list was assessed using the PIM list defined in the COMPASS (COMPrehensive geriatric AsseSSment and multidisciplinary team intervention for hospitalized older adults) study (34). The burden of comorbidity was quantified using the Charlson comorbidity index (CCI), which contains 19 categories of chronic diseases (35). Muscle strength was assessed by the handgrip strength in the dominant hand with the patient in the sitting position with elbows flexed at 90° or in the supine position if patients were unable to maintain a sitting position. Handgrip strength was measured using a Jamar Plus + Digital Hand Dynamometer (Patterson Medical). Handgrip strength was measured twice and the maximum value was used for analysis (36, 37). Cognitive function was measured using the Korean version of the Mini-Mental State Examination-2 (38). Depression was assessed using the short form of the Korean Geriatric Depression Scale (39). Nutritional status was defined according to the MNA scores as normal (> 24), risk of malnutrition (17–24), and malnutrition (< 17) (40). Nutritional status was also categorized using the biochemical marker, albumin, according to the serum albumin level: normal (> 3.9 g/dL), risk of malnutrition (3.5–3.9 g/dL), and malnutrition (< 3.5 g/dL) (41).

### 2.3. Outcome

The primary outcome was that patients could not be discharged to their own homes. The secondary outcomes were overall all-cause mortality, 3-month all-cause mortality after discharge, and incidence of geriatric syndrome during hospitalization. Incidence of geriatric syndrome was defined as a composite outcome of delirium, falls, and newly developed or worsening pressure sores during hospitalization. Delirium was defined as newly administered medications for delirium symptom control (e.g., haloperidol, quetiapine, or olanzapine) or consultation with the neuropsychiatric department for delirium. Falls were detected using a formal mandatory report for falls in the nursing department. Pressure sores were evaluated using a weekly report for pressure sores, which contained information on the site and grade of the pressure sores documented by the nursing department. Mortality data up to December 12, 2020 were obtained from the Ministry of Security and Public Administration.

### 2.4. Statistical analysis

Statistical analyses were performed using PASW Statistics software (version 25.0; SPSS Inc.) and MedCalc (MedCalc Software Ltd.). Continuous variables are expressed as means (standard deviations, SDs), and qualitative variables are presented as counts and percentages. Statistical differences were assessed using the one-way analysis of variance or Pearson’s chi-square test. The relationship between malnutrition assessed using the MNA or albumin levels and adverse outcomes was determined using (a) age, sex, and body mass index (BMI); (b) age, sex, BMI, and admission site; and (c) a fully adjusted logistic regression model for the relevant prognostic variables. Full adjustment was conducted for age, sex, BMI, admission site, CCI, hemoglobin levels, and creatinine levels. Hazard ratios for all-cause mortality according to nutritional status assessed by the MNA and serum albumin level were analyzed using Cox proportional hazards models. Kaplan–Meier analysis was used for survival curves, and log-rank tests were used to assess significance. We determined the model’s predictive value for 3-month mortality after discharge with two malnutrition assessment methods (albumin levels and the MNA) by comparing the areas under the receiver operating characteristic (ROC) curve.

## 3. Results

**FIGURE 1:**
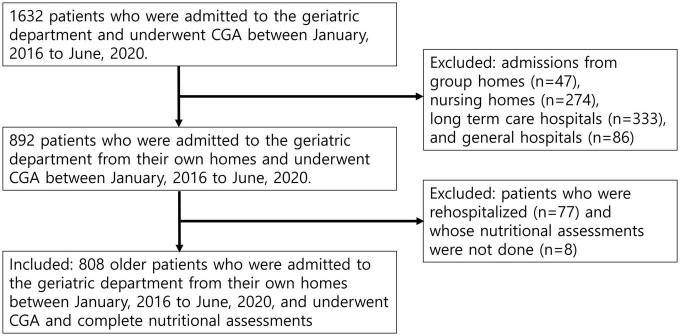
Flow of patients through the study. The study participants consisted of 808 older hospitalized patients admitted from their own homes to the geriatric department ward and underwent comprehensive geriatric (CGA) and complete nutritional assessments.

During the study period, 1,632 patients (aged ≥ 65 years) were admitted to the Geriatric Center and underwent CGA. Among them, 740 patients were admitted from centers other than their homes (47 from group homes, 274 from nursing homes, 333 from long-term care hospitals, and 86 from hospitals). After excluding 77 rehospitalizations and 7 patients for whom nutritional assessments were not performed, 808 patients were included in the analysis (Figure 1).

The mean age of the patients was 82.8 years (SD, 6.70) and 45.9% were male. The mean length of hospital stay was 11.3 days (SD, 21.9). Among the patients, 77.8% were admitted through emergency room, and they were followed for an average of 452.5 days (interquartile range, IQR, 148.25–919.75). Among them, 43 patients (5.3%) had in-hospital mortality, 160 (19.8%) had 3-month mortality, and 417 patients (51.6%) had all-cause mortality. One hundred and ninety-eight (24.5%) patients experienced geriatric syndromes of delirium (*n* = 66, 8.2%), falls (*n* = 17, 2.1%), and newly developed or worsening pressure sores (*n* = 134, *n* = 16.6%). Nineteen patients experienced two types of geriatric syndrome.

We analyzed the relationship between general patient characteristics, including patients’ demographic data, laboratory data, and CGA components, according to the nutritional status assessed by the MNA (Table 1). Patients who were malnourished were predominantly older; female; admitted through the emergency room; and had a higher risk of pressure sores and falls, ADL dependency or instrumental activities of daily living (IADL); lower Mini-Mental State Examination (MMSE) score; higher Korean version of the short form of the Geriatric Depression Scale (SGDS-K) score; lower grip strength; and shorter mid-arm circumference (MAC) and calf circumference (CC). Patients with poor nutrition tended to have lower serum albumin, protein, cholesterol, and hemoglobin levels (Table 1).

**TABLE 1:** General patient characteristics and results of the comparison of patients’ nutritional status assessed by MNA.

	Total (*n* = 808)	Normal (*n* = 98, 12.1%)	Risk of malnutrition (*n* = 284, 35.1%)	Malnutrition (*n* = 426, 52.7%)	*p*
Age (years)	82.8 (6.7)	81.7 (6.2)	81.6 (6.4)	83.9 (6.8)	<0.001
Sex (male)	371 (45.9%)	59 (60.2%)	120 (42.3%)	192 (45.1%)	0.008
BMI (kg/m^2^)	21.6 (4.5)	26.3 (3.9)	23.4 (3.5)	19.4 (3.8)	<0.001
Admission site (emergency room)	629 (77.8%)	71 (72.4%)	204 (71.8%)	354 (83.1%)	0.001
Length of hospital stay	11.6 (21.9)	8.6 (13.9)	10.1 (9.9)	13.4 (28.2)	0.052
Hendrich scale	6.2 (2.8)	4.7 (2.6)	5.7 (2.6)	6.8 (2.8)	<0.001
Braden scale	16.2 (3.9)	19.2 (2.9)	17.7 (3.4)	14.5 (3.5)	<0.001
CCI (score)*	2.4 (1.9)	2.1 (1.8)	2.4 (2.0)	2.4 (1.9)	0.289
ADL (score)^†^	65.4 (39.8)	97.2 (8.7)	85.5 (26.5)	44.6 (39.8)	<0.001
IADL (score)^‡^	3.1 (2.6)	5.3 (1.8)	4.5 (2.4)	1.7 (2.1)	<0.001
SGDS-K (score)^||^	5.5 (3.6)	3.6 (2.7)	5.9 (3.6)	6.8 (3.9)	<0.001
MMSE (score)^§^	11.3 (10.0)	21.5 (5.5)	16.1 (8.3)	5.7 (8.0)	<0.001
Grip strength (kg)^¶^	16.7 (8.0)	22.4 (8.7)	17.0 (6.8)	13.0 (6.8)	<0.001
MAC (cm)**	23.1 (3.6)	26.2 (2.9)	24.4 (2.8)	21.6 (3.4)	<0.001
CC (cm)**	28.1 (4.4)	33.2 (3.8)	30.1 (3.1)	25.7 (3.5)	<0.001
Number of medications	10.0 (5.3)	10.5 (6.3)	9.7 (5.2)	10.1 (5.0)	0.449
Number of PIMs	0.27 (0.54)	0.30 (0.56)	0.24 (0.51)	0.28 (0.56)	0.614
Albumin (g/dL)	3.2 (0.6)	3.6 (0.5)	3.4 (0.6)	3.0 (0.6)	<0.001
Protein (g/dL)	6.4 (0.9)	6.6 (0.9)	6.5 (0.9)	6.3 (0.9)	<0.001
Cholesterol (mg/dL)^‡^	133.7 (43.4)	139.3 (42.7)	137.8 (42.8)	129.7 (43.7)	0.020
Hemoglobin (g/dL)	11.5 (2.2)	12.1 (1.9)	11.5 (2.1)	11.3 (2.3)	0.002
Creatinine (mg/dL)	1.5 (1.4)	1.2 (0.5)	1.5 (1.3)	1.5 (1.6)	0.158

*n = 801, ^†^*n* = 805, ^‡^*n* = 806, ^§^*n* = 618, ^||^*n* = 251, ^¶^*n* = 460, **n = 787.

ADL, activities of daily living; CC, calf circumference; CCI; Charlson’s comorbidity index; IADL, instrumental activities of daily living; MAC, mid arm circumference; MMSE, mini-mental status examination; SGDS-K, short form of the Korean Geriatric Depression Scale; PIM, potentially inappropriate medication.

Statistical difference was assessed by one-way analysis of variance or Pearson’s chi-squared test.

The relationship between malnutrition status assessed using the MNA and serum albumin levels and outcomes was analyzed using multilevel multiple logistic regression. The fully adjusted odds ratios (ORs) for discharge to nursing homes or long-term care hospitals were 4.271 (95% CI: 1.499–12.170, *P* = 0.042) for the risk of malnutrition and 5.822 (95% CI: 2.092–16.199, *P* = 0.001) for malnutrition compared to normal nutritional status assessed by the MNA (Table 2). However, nutritional status assessed by serum albumin level could not predict discharge to nursing home or long-term care hospitals with ORs of 1.751 (95% CI: 0.771–0.377, *P* = 0.181) for risk of malnutrition and 2.164 (95% CI: 0.993–4.715, *P* = 0.052) for malnutrition compared to normal nutritional status (Table 3). In the fully adjusted model, only malnutrition status assessed by the MNA showed statistical significance in predicting geriatric syndrome, with an OR of 2.069 (95% CI: 1.007–4.249, *P* = 0.0480) (Table 2). Malnutrition status assessed by both the MNA and serum albumin level could predict 3-month all-cause mortality after discharge with ORs of 3.519 (95% CI: 1.254–9.872, *P* = 0.017) and 3.848 (95% CI: 1.465–10.105, *P* = 0.006), respectively (Tables 2, 3). Both risk of malnutrition and malnutrition assessed by the MNA and serum albumin level was associated with all-cause mortality (Table 4).

**TABLE 2:** Odds ratios for incident geriatric syndrome, discharge location and 3-months mortality according to nutritional status assessed by MNA.

	Model 1*	Model 2^†^	Model 3^‡^
**Geriatric syndrome**			
Normal (reference)	Reference	Reference	Reference
Risk of malnutrition	1.210 (0.600–2.442) *P* = 0.595	1.196 (0.594–2.408) *P* = 0.615	1.136 (0.553–2.331) *P* = 0.728
Malnutrition	2.299 (1.140–4.633) ***P* = *0.020***	2.268 (1.122–4.585) ***P* = *0.023***	2.069 (1.007–4.249) ***P* = *0.048***
**Discharge to nursing home or long-term care hospital**			
Normal (reference)	Reference	Reference	Reference
Risk of malnutrition	4.277 (1.549–11.805) ***P* = *0.005***	4.131 (1.504–11.346) ***P* = *0.006***	4.271 (1.499–12.170) ***P* = *0.007***
Malnutrition	6.644 (2.432–18.147) ***P* < *0.001***	6.587 (2.401–18.073) ***P* < *0.001***	5.822 (2.092–16.199) ***P* = *0.001***
**3-months mortality**			
Normal (reference)	Reference	Reference	Reference
Risk of malnutrition	3.685 (1.260–10.782) ***P* = *0.017***	3.573 (1.223–10.438) ***P* = *0.020***	2.468 (0.806–7.556) *P* = 0.114
Malnutrition	3.931 (1.421–10.875) ***P* = *0.008***	3.842 (1.388–10.635) ***P* = *0.010***	3.519 (1.254–9.872) ***P* = *0.017***

Data are presented as odds ratio (95% confidence interval).

*Adjusted by age, sex, body mass index.

^†^Adjusted by age, sex, body mass index and admission site.

^‡^Adjusted by age, sex, body mass index, admission site, CCI, hemoglobin and creatinine.

Values in bold and italic indicate statistical significance.

**TABLE 3:** Odds ratios for incident geriatric syndrome, discharge location, and 3-months mortality according to nutritional status assessed by albumin.

	Model 1*	Model 2^†^	Model 3^‡^
**Geriatric syndrome**			
Normal (reference)	Reference	Reference	Reference
Risk of malnutrition	1.749 (0.884–3.460) *P* = 0.108	1.014 (0.969–1.061) *P* = 0.553	1.713 (0.853–3.440) *P* = 0.130
Malnutrition	1.964 (1.046–3.689) ***P* = *0.036***	1.657 (0.872–3.148) *P* = 0.123	1.753 (0.899–3.419) *P* = 0.099
**Discharge to nursing home or long-term care hospital**			
Normal (reference)	Reference	Reference	Reference
Risk of malnutrition	1.652 (0.743–3.675) *P* = 0.218	1.601 (0.718–3.573) *P* = 0.250	1.751 (0.771–3.77) *P* = 0.181
Malnutrition	2.630 (1.267–5.457) ***P* = *0.009***	2.065 (0.978–4.362) *P* = 0.057	2.164 (0.993–4.715) *P* = 0.052
**3-months mortality**			
Normal (reference)	Reference	Reference	Reference
Risk of malnutrition	1.599 (0.552–4.403) *P* = 0.402	1.379 (0.481–3.952) *P* = 0.549	1.443 (0.484–4.306) *P* = 0.511
Malnutrition	4.338 (1.694–11.113) ***P* = *0.002***	4.006 (1.556–10.317) ***P* = *0.004***	3.848 (1.465–10.105) ***P* = *0.006***

Data are presented as odds ratio (95% confidence interval).

*Adjusted by age, sex, body mass index.

^†^Adjusted by age, sex, body mass index and admission site.

^‡^Adjusted by age, sex, body mass index, admission site, CCI, hemoglobin and creatinine.

Values in bold and italic indicate statistical significance.

**TABLE 4:** Hazard ratios for all-cause mortality according to nutritional status assessed by albumin and MNA.

	Model 1*	Model 2^†^	Model 3^‡^
**Albumin**			
Normal (reference)	Reference	Reference	Reference
Risk of malnutrition	1.682 (1.062–2.663) ***P* = *0.027***	1.605 (1.011–2.547) ***P* = *0.045***	1.638 (1.023–2.623) ***P* = *0.040***
Malnutrition	2.680 (1.759–4.082) ***P* < *0.001***	2.588 (1.694–3.953) ***P* < *0.001***	2.632 (1.702–4.071) ***P* < *0.001***
**MNA**			
Normal (reference)	Reference	Reference	Reference
Risk of malnutrition	1.831 (1.172–2.858) ***P* = *0.008***	1.832 (1.175–2.858) ***P* = *0.008***	1.599 (1.019–2.509) ***P* = *0.041***
Malnutrition	2.693 (1.729–4.193) ***P* < *0.001***	2.669 (1.714–4.157) ***P* < *0.001***	2.341 (1.489–3.381) ***P* < *0.001***

Data are presented as hazard ratio (95% confidence interval).

*Adjusted by age, sex, body mass index.

^†^Adjusted by age, sex, body mass index and admission site.

^‡^Adjusted by age, sex, body mass index, admission site, CCI, hemoglobin and creatinine.

Values in bold and italic indicate statistical significance.

**FIGURE 2:**
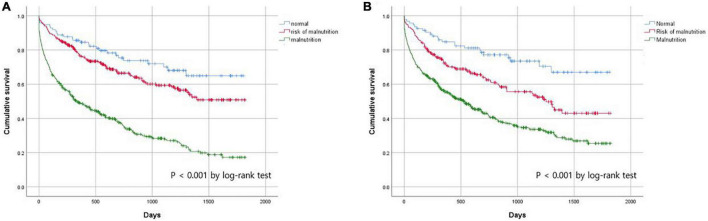
Cumulate survival rate according to nutritional status assessed by **(A)** MNA and **(B)** albumin.

To assess the prognostic utility of malnutrition assessed by the MNA and serum albumin levels, we conducted a Kaplan–Meier analysis (Figure 2). Malnutrition assessment according to the MNA and serum albumin levels successfully predicted all-cause mortality; *post hoc* analysis to compare normal vs. risk of malnutrition, normal vs. malnutrition, and risk of malnutrition vs. malnutrition showed that they were all statistically significant in both assessments.

**FIGURE 3:**
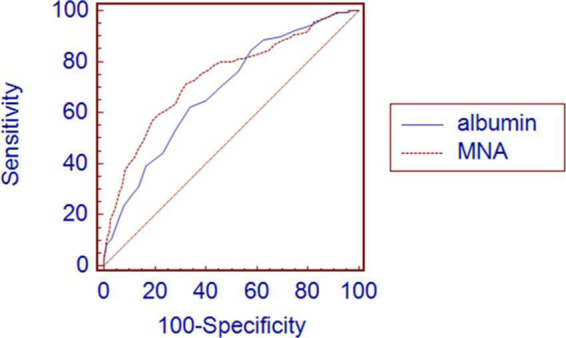
Comparison of area under receiver operating characteristic curve between albumin and MNA for 3-month all-cause mortality after discharge. Graph shows higher AUC in MNA 0.739 (0.707–0.769) than albumin 0.686 (0.653–0.718) with significant *P*-value of 0.034.

The MNA predicted 3-month all-cause mortality more accurately than the serum albumin levels according to the comparison of the area under the ROC curve (AUC). The AUCs for the predictive model according to the MNA and albumin levels were 0.739 (95% CI: 0.707–0.769) and 0.686 (95% CI: 0.653–0.718), respectively. The pairwise comparison of the AUC was statistically significantly different between the MNA and serum albumin levels (*P* = 0.034) (Figure 3).

## 4. Discussion

Our study shows that malnutrition status defined by the MNA was significantly associated with adverse outcomes in older patients hospitalized in acute geriatric centers. Older inpatients with malnutrition were five times more likely to be discharged to nursing homes or long-term care hospitals and three times more likely to die within 3 months. Additionally, their chance of developing geriatric syndrome during hospitalization more than doubled.

Frailty, as a reflection of decreased physiological reserve, is closely associated with biological age (42), concurrent medical conditions, morbidity, and decreased survival in older adults (43). In frailty assessments, parameters reflecting nutritional status are commonly included, as malnutrition is considered a key factor in the progression of frailty (11, 12). The addition of a stressor event such as pneumonia or urinary tract infection to a frail older person with impairment of balance or cognition explains the geriatric syndromes of falls and delirium, respectively, as consequences of the loss of homeostatic reserve (44). Unintentional weight loss, a representative criterion for the frailty phenotype model (11), is a major risk factor for pressure sore development (14).

There are various definitions for aging in place, but it generally refers to the phenomenon of older adults that remain living within their communities with some level of independence, rather than in residential care (45). One of the biggest threats to aging in place is that older adults become ADL-dependent due to functional decline after acute disease. In our study, there was a significant difference in the ADL score according to nutritional status. Therefore, it is understandable that nutritional status can influence the discharge location.

In the past, serum albumin was widely used as an indicator of malnutrition in older patients (46, 47). It is well known that serum albumin levels are an independent risk factor for all-cause mortality in older adults (48). Our models also showed that serum albumin levels could predict 3-month all-cause mortality after discharge. However, nutritional status evaluated using the MNA showed a significant association with discharge location and geriatric syndrome, whereas nutritional status evaluated using serum albumin had no significant association. This may be because the MNA has the advantage of predicting functional decline (49, 50). The fact that the MNA is a multidimensional tool that includes general assessment, dietary assessment, and anthropometric assessment can make this prediction possible (23). Representatively, CC included in the MNA is used to screen for sarcopenia (51, 52), a major cause of functional decline in older adults (53, 54). Because functional decline is closely associated with mortality (55, 56), it is reasonable that the MNA is a better predictor of post-discharge mortality than serum albumin levels. Recent studies showed that MNA is also useful to detect frailty status in older adults (26, 57).

From our observations, we posit that nutritional status should be considered when establishing a protocol for treating acute hospitalized older patients to prevent adverse outcomes, such as death and nursing facility admission. With nutrition comprising the core element of multidimensional frailty preventative measures, it is necessary to maximize the potential benefits of nutritional support programs; further nutritional intervention studies on acute-hospitalized older patients are warranted. In our study group, cut-off points to predict 3 months mortality was MNA score ≤ 14 according to highest Youden index, with a sensitivity of 71.5% and specificity of 67.5%, while positive predictive value and negative predictive value were 34.14 and 90.97%, respectively. The cut-off point would be useful to identify the high-risk patients who can benefit from nutritional support program.

This study has several strengths. First, the analysis of the impact of malnutrition on clinical outcomes included the discharge location, and statistically significant results were obtained even after adjusting for multiple covariates. Second, by analyzing the impact of nutritional status in acute geriatric patients, malnutrition was identified as a target for interventional studies to improve the clinical outcomes of hospitalized older adults. This study also has some limitations. First, because this study was conducted in inpatients at a university hospital, it is difficult to support the generalization of the study. Second, in some cases, the various circumstances of caregivers may influence the decision of discharge location for older patients. However, factors related to caregivers were not included in this analysis. Third, serum albumin has a limitation in that it is difficult to accurately evaluate the nutritional status of patients in the acute phase. Current paper recommends that serum albumin must be correctly recognized as an inflammatory marker associated with “nutritional risk” in nutrition assessment and should not be inappropriately interchanged with concept of malnutrition (58). However, serum albumin was traditionally considered a useful biochemical laboratory value in nutritional assessment and currently there is a lack of biomarkers widely used to replace it. Therefore, in our retrospective design study, serum albumin was used as a clinically widely used biomarker measured in all study participants.

In conclusion, nutritional status evaluated using the MNA was an independent predictor of various negative outcomes among older hospitalized patients. Poor nutritional status assessed by serum albumin levels, the most widely used biochemical marker, could predict mortality, but not geriatric syndrome or discharge location, which might reflect the patients’ functional decline. As a multidimensional tool, the MNA needs to be used more actively for the nutritional assessment of geriatric patients.

## Data availability statement

The raw data supporting the conclusions of this article will be made available by the authors, without undue reservation.

## Ethics statement

The studies involving human participants were reviewed and approved by the Institutional Review Board of Seoul National University Bundang Hospital. Written informed consent for participation was not required for this study in accordance with the national legislation and the institutional requirements.

## Author contributions

K-IK: conceptualization and funding acquisition. J-YC: data curation and formal analysis. M-GK, J-YC, H-JY, S-YP, YK, JK, S-WK, and C-HK: investigation and project administration. K-IK and J-YC: methodology and validation. K-IK and C-HK: supervision. M-GK and J-YC: visualization, roles, and writing—original draft. M-GK, J-YC, and K-IK: writing—review and editing. All authors contributed to the article and approved the submitted version.
